# Rapid manufacturing of micro-drilling devices using FFF-type 3D printing technology

**DOI:** 10.1038/s41598-021-91149-8

**Published:** 2021-06-09

**Authors:** Sangyeun Park, Byeongjo Ko, Heewon Lee, Hongyun So

**Affiliations:** 1 Department of Mechanical Engineering, Hanyang University, Seoul, 04763 South Korea; 2 Institute of Nano Science and Technology, Hanyang University, Seoul, 04763 South Korea

**Keywords:** Mechanical engineering, Surface patterning

## Abstract

Micro-drilling devices with different blade shapes were fabricated with a rapid and facile manufacturing process using three-dimensional (3D) printing technology. The 3D-printed casting mold was utilized to customize the continuous shape of the blades without the need for expensive manufacturing tools. A computational fluid dynamics simulation was performed to estimate the pressure differences (fluidic resistance) around each rotating device in a flowing stream. Three types of blades (i.e., 45°, 0°, and helical type) were manufactured and compared to a device without blades (i.e., plain type). As a result, the device with the 45° blades exhibited the best drilling performance. At a rotational speed of 1000 rpm, the average drilling depth of the device with the 45° blades to penetrate artificial thrombus for 90 s was 3.64 mm, which was ~ 2.4 times longer than that of helical blades (1.51 mm). This study demonstrates the feasibility of using 3D printing to fabricate microscale drilling devices with sharp blades for various applications, such as in vivo microsurgery and clogged water supply tube maintenance.

## Introduction

Biomedical microdevices are widely studied for their promising and advantageous applications such as sensing^1,2^, detailed diagnosis^3^, drug delivery^1,3–7^, and microsurgery^7^. Vascular catheters^8,9^, heart sleeves^10^, medical gauzes^1^, guide wires^7,8,11^ and cannulas^1,9^ are representative examples. In particular, drill-shaped microdevices have emerged as a facile and controllable actuator to maintain the continuous flow of blood or water in circular tubes^12–14^. For example, drill-type biomedical devices are used to drill clogged tubes in the body^4,5,8,9,15^, puncture a bone^16^ and move the robot^3,8,12,15,17–19^. In these drill-type biomedical devices, the purpose and performance vary with the blade shapes. The study of blade parameters reported differences in propulsion that depend on the number of blades or angle^8,15,17^. To fabricate drill-type medical devices, various methods such as multi-jet modeling-based three-dimensional (3D) printing^4,5,20^, casting^7,21^, and surface micromachining^22^ have been investigated. Although the conventional micro fabrications could create sharp blades with precise control, the manufacturing processes are expensive, complex, and have difficulty in modifying the device parameters upon user requests. In contrast, the additive manufacturing could create objects with facile and rapid processes, however, the processes are still limited to form sharp blades which are thinner than printing resolutions. Therefore, the needs of manufacturing processes for creating sharp blades, that are facile, fast, and easy to modify, are still demanded.

As 3D printing technology has become more accessible and widely used, a new manufacturing process paradigm has emerged^23^. In case of fused filament fabrication (FFF)-type 3D printing, rapid and cost-effective fabrication using ecofriendly^24,25^ and biocompatible^26^ materials are achievable. Moreover, FFF-based manufacturing provides an easy and fast approach to modify device dimensions. In particular, casting methods using 3D-printed molds have been widely researched owing to their advantages and applicability to various fields with different commercial materials (filaments). In addition, recent studies have revealed the capability of 3D printing to express micropatterns, and its uses on hydrophobic surfaces^27^, microchannels^28,29^, and moisture capture^30^. In general, the quality (surface roughness) of the end products printed by FFF-type 3D printer highly depends on various printing conditions such as printing speed, nozzle size, filament thickness, printing resolution, printing angle, and extruder temperature. Although the roughness can be smoothened by a post-treatment process such as acetone fumigation, this staircase effect on the surface caused by the stacked filaments in multilayers still remains as an engineering challenge in additive manufacturing.

In this study, micro-drilling devices (MDDs) with micropatterns are fabricated and demonstrated using FFF-type 3D printing methods, which easily modify the device features with simple and rapid manufacturing processes. Interestingly, the staircase problem (i.e., drawback of FFF-type 3D printing manufacturing) was intentionally used to create the sharp blades of the microdrill without the use of complex and expensive micromachining tools^31^. The 3D mold printed with stacked filaments could form blades of various shapes in microdrill devices. In addition, the shape of the blades can be adjusted depending on the stack angle and stack layer of the filaments. In particular, the sharp edge of blades thinner than the layer height was successfully achieved by the casting method. Computational fluid dynamics (CFD) simulations and drilling experiments were conducted to characterize and compare the performance of microdrills with four different shapes. The microdrills manufactured in this study have various biomedical applications such as removing blood clots in blood vessels or environmental applications such as piercing the blocked water supply tubes, serving as catheters or drilling robots, respectively.

## Design and manufacturing process

**Figure 1:**
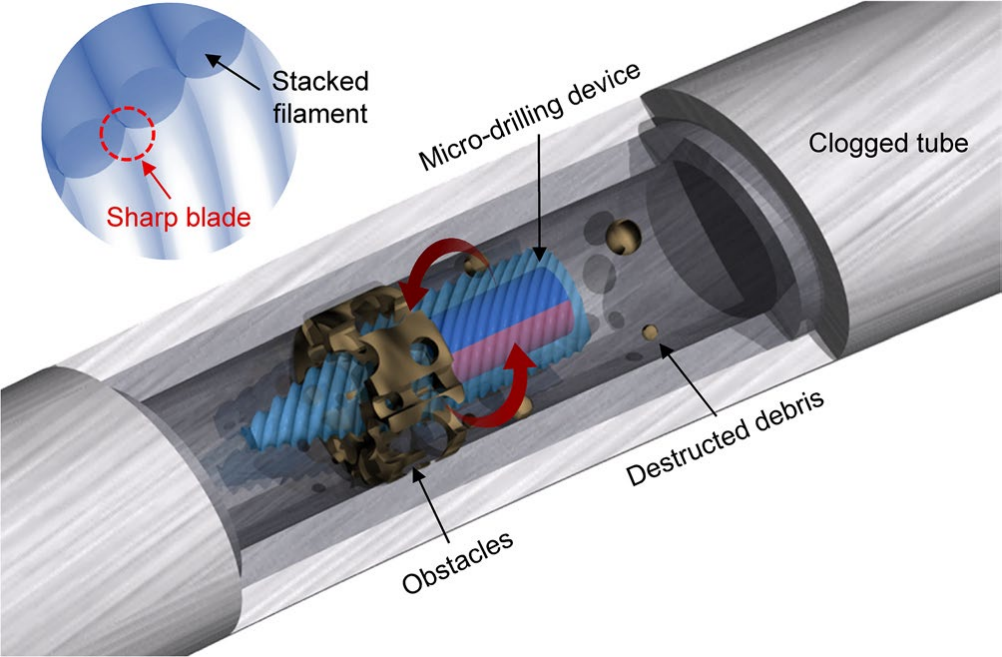
Schematic of the micro-drilling device which destroys the clogged materials in a tube such as blood vessel or water supply line. Inset: formation of sharp waveform blades by the staircase effect between stacked filaments.

**Figure 2:**
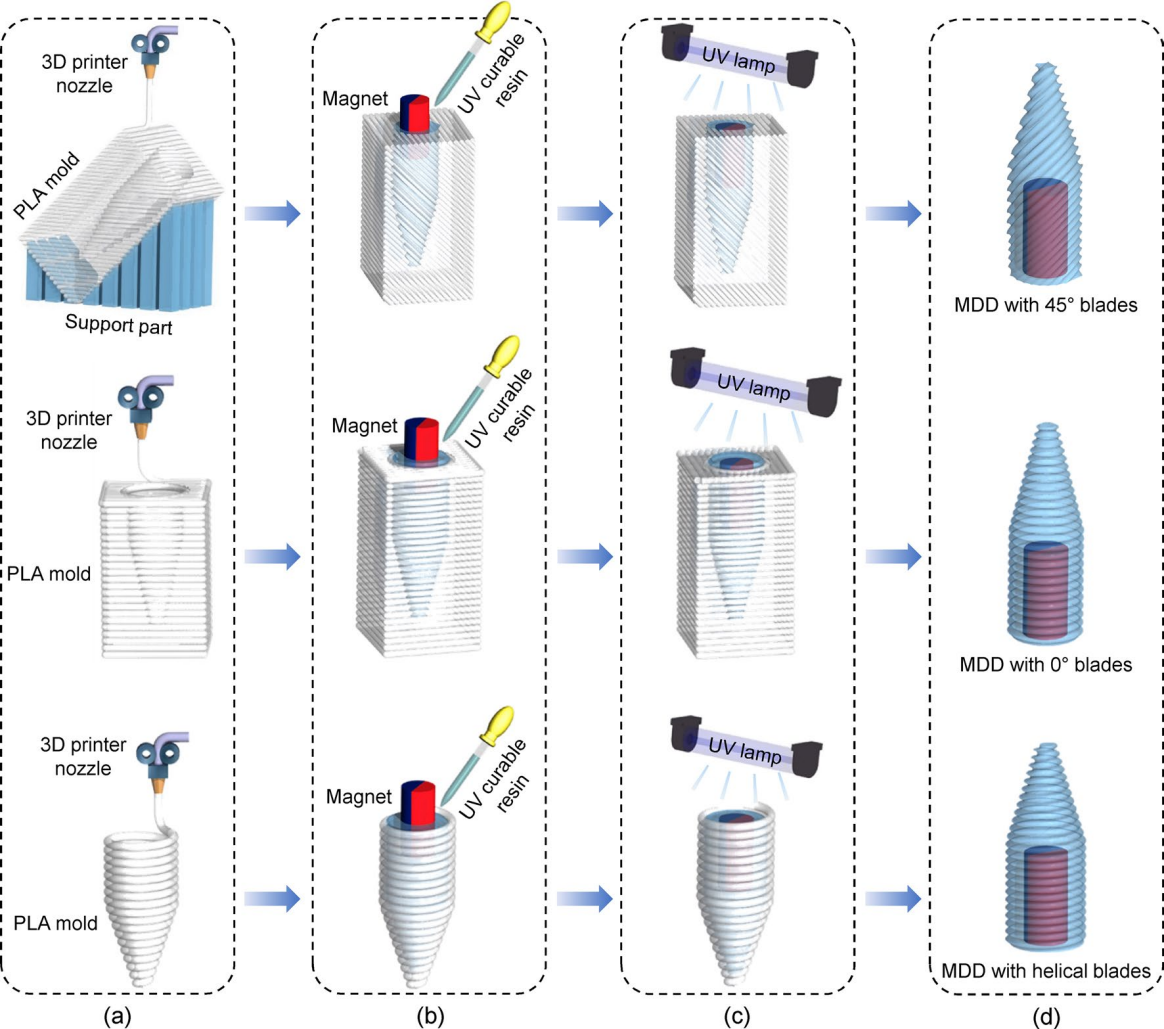
Overall fabrication process to manufacture MDDs with 45°-tilted, 0°, and helical blades. (**a**) 3D printing the PLA casting mold using FFF-type 3D printer; (**b**) Pouring the UV curable resin into the casting mold followed by degassing process, and inserting the neodymium magnet; (**c**) Curing the resin using UV lamp; (**d**) Separating the MDD from the casting mold by soaking in acetone.

Figure 1 shows a schematic of the MDD that can be actuated (rotated) by magnetic fields. As the MDD rotates, the device moves in a narrow tube and destroys the obstacles clogged in the tube, as shown in Fig. 1. Using the structural characterization of FFF-type 3D printing methods, MDDs with four different blade shapes were fabricated and compared. It should be noted that the blades with sharp waveform were obtained by casting the empty space caused by the staircase effect between stacked filaments, as shown in inset image in Fig. 1. In this study, four types of 3D-printed MDDs were tailored, (i.e., MDDs with 45°, 0°, helical blades, and without blades). Figure 2 shows the overall manufacturing process of the MDDs with 45°, 0°, and helical blades. It is noteworthy that the angle between the base of the device and the blade can be easily controlled by adjusting the printing angle during the manufacturing process. By changing the printing mode, an MDD with helical blades was also manufactured. In addition, to demonstrate the role of blades in the removal of residues in clogged tubes, an MDD without blades was fabricated, and the drilling performance was compared with that of other MDDs. The main body of the MDDs was designed in a bullet shape to contain a cylindrical magnet inside the body and to reduce the drag force in the stream simultaneously^32^.

For the manufacturing of MDDs with 45°-tilted blades and 0°-tilted blades, polylactic acid (PLA) filaments (natural type, ultra-pure grade, Ø1.75 mm) were extruded through the FFF-type 3D printer (GUIDER IIS, FlashForge) to create the casting mold with rough surfaces. To manufacture the helical blades, an advanced 3D printer (3DWOX DP200, Sindoh) was used to create the PLA casting mold. To create the MDD without blades (i.e., plain type), a casting mold without blade patterns was prepared using the double casting method. The 3D printing conditions used to manufacture each MDD are summarized in Table 1. The process consists of four steps: printing the PLA casting mold (Fig. 2a), pouring the ultraviolet (UV) curable resin with a magnet (Fig. 2b), curing the resin under UV light (Fig. 2c), and separating the MDDs from the casting mold (Fig. 2d). The only difference in the manufacturing of MDDs with 45°, 0°, and the MDD with helical blades was the first step (i.e., printing the PLA casting mold). To prepare the PLA casting mold that produces the 45°-tilted blades, the PLA mold was printed on the support part at an angle of 45° such that the stacked filaments could create a 45°-tilted pattern on the bullet-shaped body of MDD. To form a casting mold that generates helical blades, the calibration option of the printer (“spiralize the outer contour” under “shape error correction” option) was used to continuously print filaments, as shown in Fig. 2a. After making the PLA casting mold, the UV-curable resin (Sun Drop, hard type, PADICO) was poured into the 3D-printed PLA casting mold and degassed in a vacuum chamber for 2 h. To rotate the MDD under magnetic fields, a diametrically magnetized cylindrical neodymium magnet (1–102.25.8D, MAGNA) was inserted into the degassed resin (Fig. 2b). The resin was fully cured for 15 min using a UV lamp (365 nm, SSUV3365, Skycares), as shown in Fig. 2c. Finally, MDDs with different blade shapes were separated from the PLA casting mold by soaking them in an acetone bath for one day (Fig. 2d).

**Table 1:** Printing conditions and parameters of 3D printer used in this study.

	45°	0°	Helical	Plain
Printing speed	60 mm/s	60 mm/s	60 mm/s	60 mm/s
Nozzle travel speed	80 mm/s	80 mm/s	80 mm/s	80 mm/s
Nozzle diameter	0.4 mm	0.4 mm	0.4 mm	0.4 mm
Extruder temperature	200 °C	200 °C	200 °C	230 °C
Platform temperature	40 °C	40 °C	40 °C	115 °C
Layer height	0.4 mm	0.4 mm	0.4 mm	0.4 mm
Printing angle	45°	0°	–	0°
Shape error correction	–	–	Spiralize outer contour	–
Material	PLA	PLA	PLA	ABS

**Figure 3:**
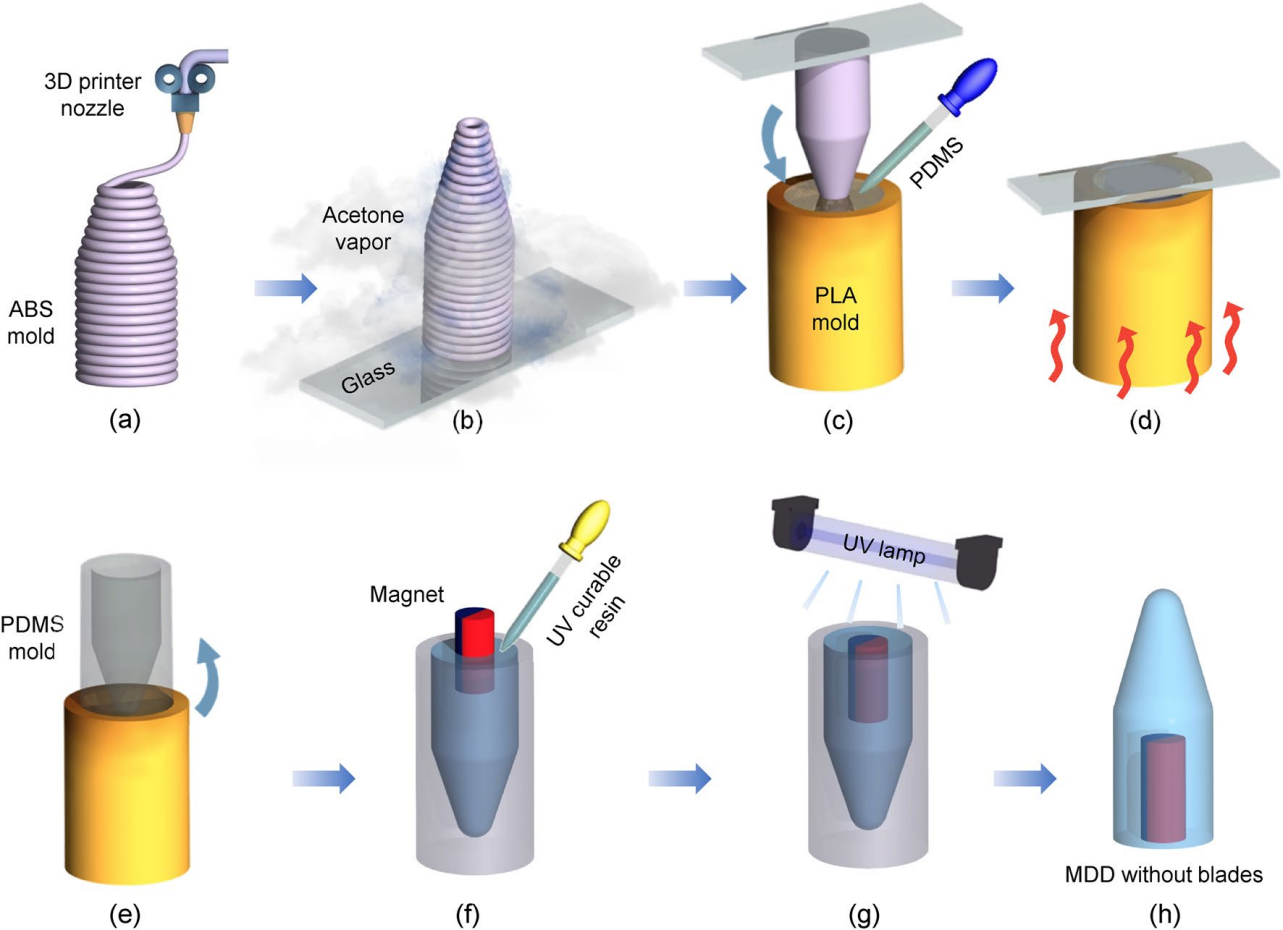
Manufacturing process for the plain-type MDD without blades. (**a**) 3D printing the ABS casting mold with 0° blades using FFF-type 3D printer; (**b**) Polishing the rough mold surface using cold acetone vapor fumigation process, (**c**) Inserting the polished ABS mold into uncured PDMS mixture and degassing PDMS; (**d**) Curing the PDMS mixture; (**e**) Separating the PDMS mold; (**f**) Pouring the UV curable resin into the PDMS casting mold followed by degassing process, and inserting the neodymium magnet; (**g**) Curing the resin using UV lamp; (**h**) Separating the MDD from the casting mold.

To demonstrate the effect of different blade shapes, the MDD without blades (plain type) was fabricated. Figure 3 shows the manufacturing process for the plain-type MDD using a double-casting process. As a first step, the casting mold for the MDD with 0° blades was printed using an acrylonitrile butadiene styrene (ABS) filament (Ø1.75 mm), as shown in Fig. 3a. Because ABS melts in acetone, the rough surface of the ABS casting mold was polished by acetone fumigation (Fig. 3b). In this process, cold acetone vapor treatment was performed for 90 min because the hot vapor treatment might create uneven surfaces. The cold vapor treatment yielded a smooth casting mold surface^33–35^. Then, a reversed mold composed of polydimethylsiloxane (PDMS) was created, as shown in Fig. 3c–e. During this process, the PDMS mixture (prepolymer: curing agent = 10:1, Sylgard 184 Silicone Elastomer Kit, Dow Corning) was poured into the cylindrical PLA mold, degassed in a vacuum chamber for 2 h, and baked at 70 °C for 5 h in an oven to cure the PDMS. The UV-curable resin was poured into the PDMS casting mold and degassed for 2 h (Fig. 3f). After inserting the neodymium magnet and UV curing for 15 min (Fig. 3g), the plain-type MDD without blades was separated from the PDMS casting mold, as shown in Fig. 3h.

## Results and discussion

**Figure 4:**
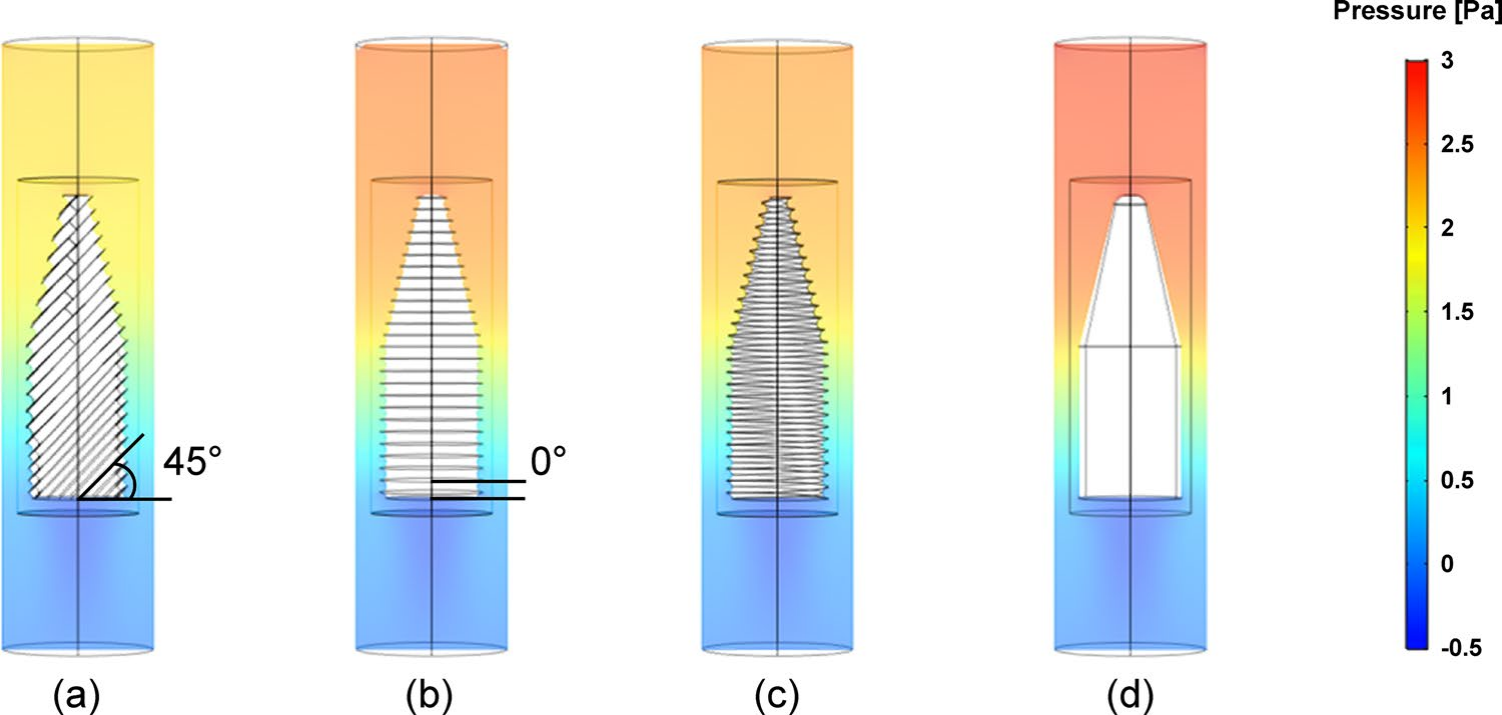
Simulated pressure distribution in the water stream with MDDs with (**a**) 45°-tilted blades, (**b**) 0° blades, (**c**) helical blades, and (d) without blades (plain type).

To compare the effect of each blade shape on the moving (swimming) performance in a flowing stream, a CFD simulation was carried out using COMSOL Multiphysics, as shown in Fig. 4. The simulation conditions are presented in Table 2. When the MDD was rotating at the center of a tube filled with water, the pressure difference between the top head and bottom tail of the MDD was calculated; they are listed in Table 3. Results show that the smallest pressure difference (1.936 Pa) and drag force (2.169 × 10^–5^N) were observed in the MDD with 45°-tilted blades, whereas the largest pressure difference (2.694 Pa) and drag force (3.135 × 10^–5^N) appeared in the MDD without blades. This suggests the considerable effect of MDD surface pattern (blade) and the bullet-shaped body on the resistance to flow during the drilling operation. Therefore, the MDD with 45°-tilted blades is expected to receive less fluidic resistance compared to the other types of MDDs.

**Table 2:** Computational conditions and dimensions of domain for CFD simulation.

Inlet velocity	Rotational speed	Reynolds number	Wall	Drill radius	Drill length	Pipe radius	Pipe length
0.01 m/s	180 rpm	49.65	No-slip	1.7 mm	10 mm	2.5 mm	20 mm

**Table 3:** Calculated pressure values at top head and bottom tail of each MDD and drag force.

Blade type	Top pressure [Pa]	Bottom pressure [Pa]	Difference [Pa]	Drag force [N]
45°	1.967	0.031	1.936	2.169 × 10^–5^
0°	2.445	0.034	2.411	2.649 × 10^–5^
Helical	2.279	0.030	2.249	2.681 × 10^–5^
Plain	2.707	0.013	2.694	3.135 × 10^–5^

**Figure 5:**
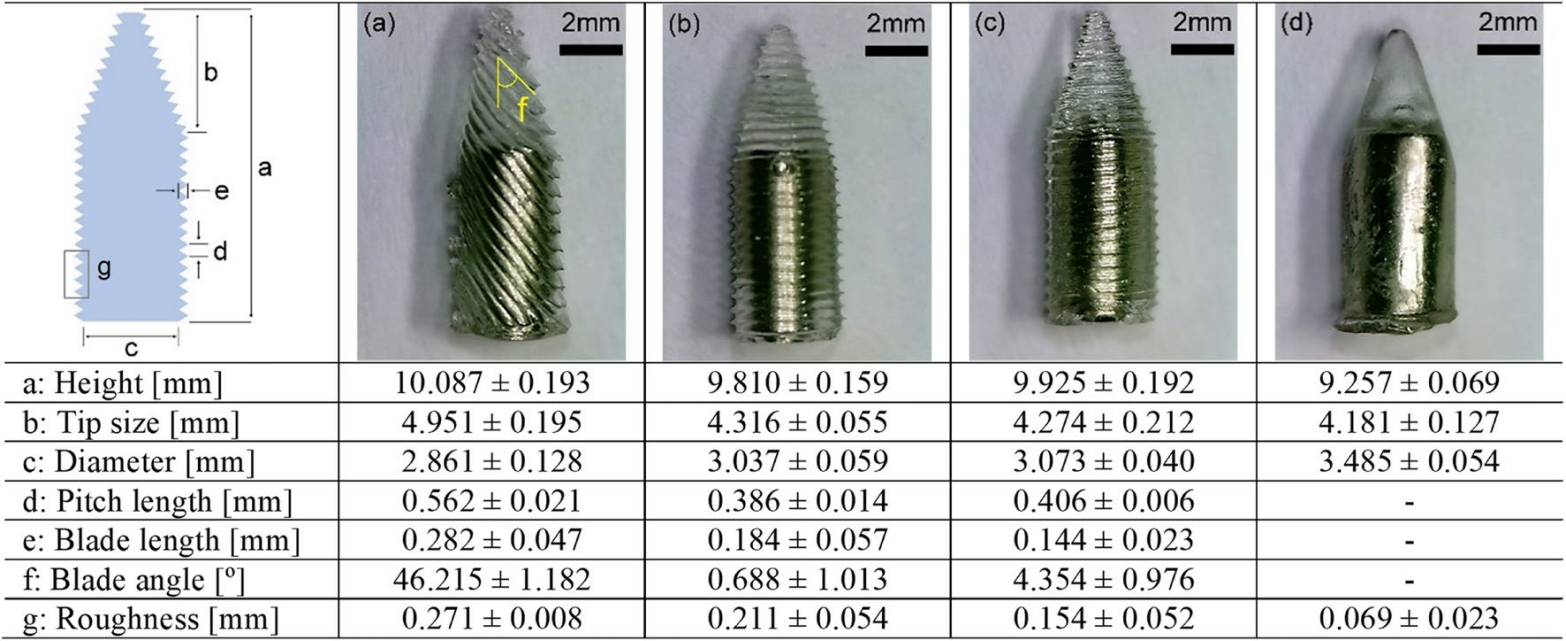
Optical image of fabricated MDDs with (**a**) 45°-tilted blades, (**b**) 0° blades, (**c**) helical blades, and (**d**) no blades (i.e., plain type) and geometrical dimensions of each MDD.

**Figure 6:**
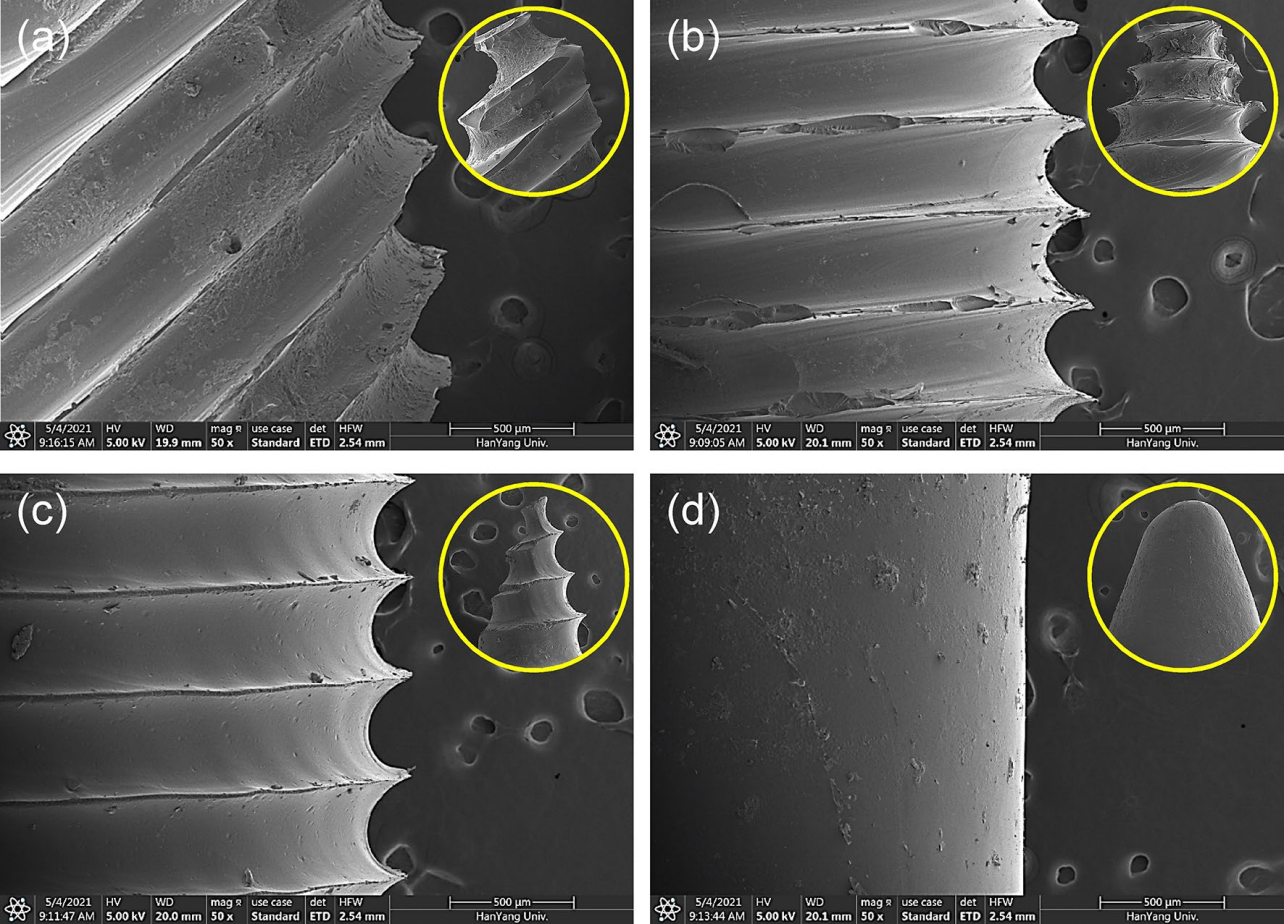
SEM images of fabricated MDDs with (**a**) 45°-tilted blades, (**b**) 0° blades, (**c**) helical blades, and (**d**) no blades (i.e., plain type). Inset: tip of each MDD.

Figure 5 shows optical images of each fabricated MDD. Each MDD has different blade shapes and a neodymium magnet inside the device. To characterize the device features, geometrical dimensions of each patterned MDD were measured. The longest blade length was 282 μm in the MDD with 45°-tilted blades. The lengths of the blades in the MDD with 0° blades and helical blades were 184 and 144 μm, respectively. Because the blade length was mostly affected by the layer height and printing angle, different blade lengths were achieved, as shown in Fig. 5. It was also noticeable that the MDD with 45°-tilted blades showed the roughest surface as the blade length was the longest compared to the others. The detailed blade features of each fabricated MDD obtained using a scanning electron microscope (SEM) are shown in Fig. 6. Figure 6a–c show the MDD with 45°, 0°, and helical blades, respectively, indicating the uniformity and sharpness of each blade. The side view of each MDD’s tip is also shown in the inset of each figure. As shown in Fig. 6d, the tip and side of the plain-type MDD were blunt (round) and smooth, respectively, as the surface patterns of the casting mold were polished by an acetone fumigation process (Fig. 3b). The height and length of each blade can notably be controlled by changing the 3D printing parameters, such as the layer height. In addition, since the main material of MDDs was the UV curable resin, the manufactured MDDs had a shore hardness of D 70 ~ 80, melting point of 160 °C, heat deflection temperature of 70 ~ 90 °C, and maximum compressive stress of ~ 5.4 MPa, which are enough mechanical properties to be used for practical applications.

**Figure 7:**
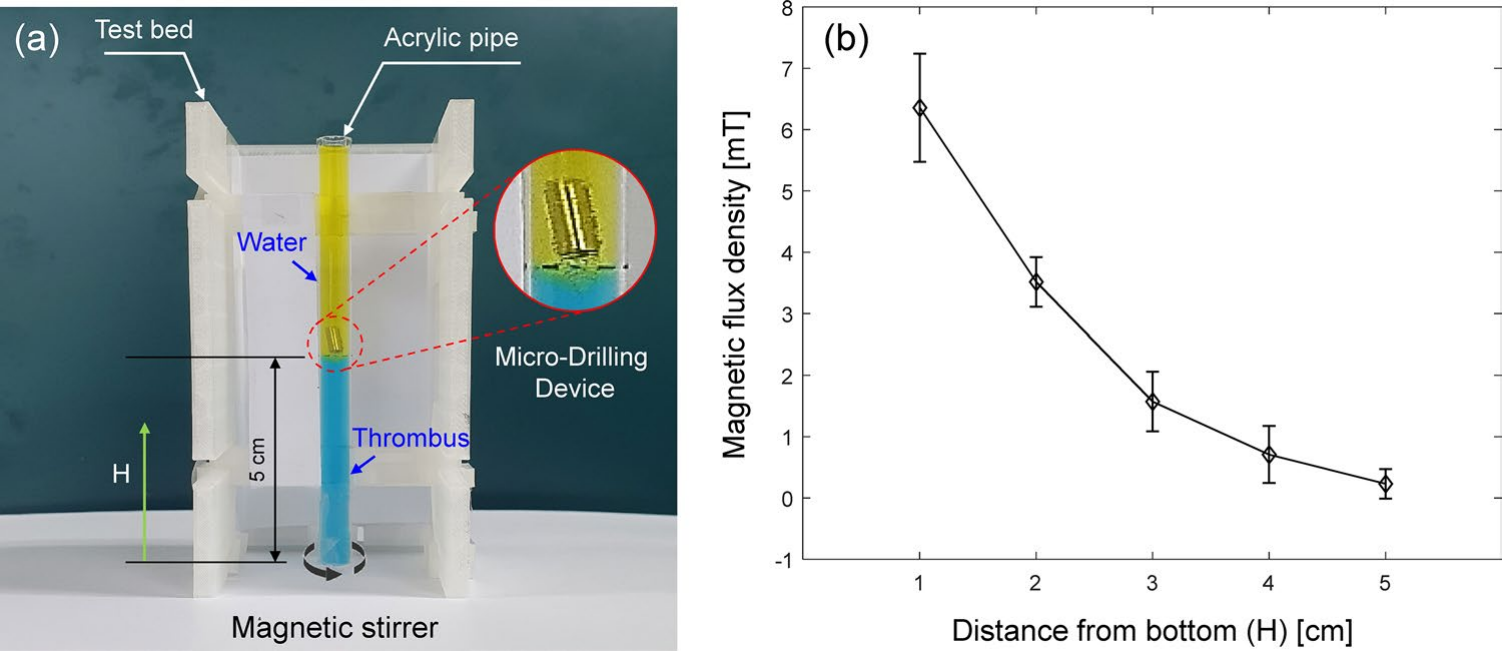
(**a**) Image of the experimental setup to demonstrate the drilling performance of the fabricated MDDs. (**b**) Magnetic flux density affecting the MDD along the distance from the bottom of the magnetic stirrer. Error bar represents the standard deviation (n = 5).

To demonstrate and compare the drilling performance of each MDD, an artificial thrombus (blood clot) was prepared using agar and inserted into a transparent pipe. The two different concentrations (0.4 and 0.6%) of agar powder were mixed with water, followed by dissolving with double boiling process at 100 °C for 3 min. The prepared mixture was dyed blue and inserted into an acrylic pipe with a length of 10 cm and a radius of 5 mm. The mixture was then hardened in the pipe at room temperature for 1 h. The rest of the acrylic pipe was filled with water, which was dyed yellow in color to visually check the penetrated depth by each MDD, as shown in Fig. 7a. The acrylic pipe was fixed in a test bed and aligned at the center of a magnetic stirrer (MaXtir 500, DAIHAN). To measure the magnetic flux density, a Gauss meter (mg-3002, Lutron) was used in this study. Figure 7b shows the measured magnetic flux density with respect to the distance from the bottom of the stirrer (measurement was performed five times at each location). As the MDD moved downward, the magnetic flux density applied on the MDD increased. The same magnetic field was applied to all MDDs for a fair comparison.

**Figure 8:**
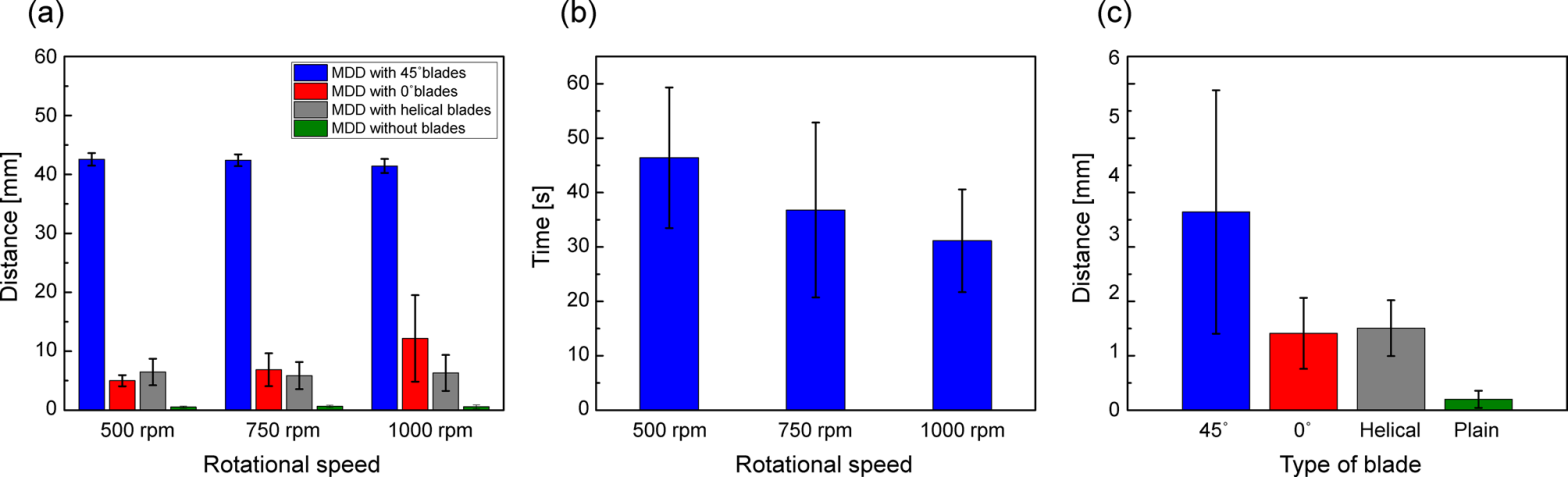
(**a**) Total distance to drill the clogged artificial thrombus (0.4% concentration) for 90 s with respect to three different rotational speed. (**b**) Required time of MDD with 45º blades to drill the 5 cm clogged artificial thrombus (0.4% concentration) with respect to the three different rotational speed. (**c**) Total penetrated distance of the clogged artificial thrombus (0.6% concentration) during a 90 s duration under the rotational speed of 1000 rpm. Total five different samples were tested three times for each result. Error bar represents the standard deviation (n = 15).

Figure 8a shows the total penetrated depth of the 5 cm deep artificial thrombus (0.4% concentration) along the pipe with respect to the rotational speed for 90 s. Total five different devices for each MDD were tested three times for each result to obtain reliable results. As a result, for all rotational speed ranges, the MDD with 45° blades exhibited the longest drilling depth to penetrate the clogged thrombus. It was noteworthy that the MDD with 45° blades fully pierced the entire 0.4% clogged thrombus during a 90 s duration at all measured rotational speeds (i.e., 500, 750, and 1000 rpm). To further characterize the drilling performance of the MDD with 45° blades with respect to the rotational speed, the required time to pierce the entire 0.4% clogged thrombus was measured again, as shown in Fig. 8b. As a result, the average drilling time of the MDD with 45° blades was shortened from 46.4 to 31.1 s when the rotational speed was increased from 500 to 1000 rpm (i.e., approximately 1.5 times faster performance). This means that the drilling time decreased as the rotational speed increased. Compared to the blade-type MDD, the plain-type MDD hardly drilled the clogged obstacles, demonstrating the role of blades in drilling performance. The difference in drilling performance with respect to the blade features was also confirmed using the penetrated depth (distance) of the 0.6% clogged thrombus, which was harder than the 0.4% thrombus. Figure 8c shows the penetrated depth of the 0.6% clogged thrombus at a rotational speed of 1000 rpm for 90 s. As a result, the average drilled distance was measured to be 3.64, 1.41, 1.51, and 0.2 mm for MDDs with 45°, 0°, helical, and without blades, respectively. The MDD with 45° blades had approximately 2.4 times longer drilling depth compared to that with helical blades. These results can be supported by CFD simulation results, showing the smallest drag force of MDD with 45° blades compared to the other types, as shown in Fig. 4. Therefore, the MDD with 45° blades had an advantageous to move forward under the same magnetic field. These results revealed that the MDD with 45° blades shows the best drilling performance, and the conventional helical blades fabricated by the 3D-printing based casting method were limited in drilling obstacles. The drilling performance of the MDD with 0° blades and helical blades did not show much difference, as shown in Fig. 8a and c. This might be because that the blade length and blade angle between the MDDs with 0° blades and helical blades were similar dimensions, thus indicating the blade length and angle played a key role in drilling performance. Unlike the 0° and helical blades (V-shaped thread), the 45° blades showed asymmetric structures (Fig. 6a) and the longest blade length. These characteristics were very similar to those of the buttress thread structure^36,37^, which likely provide low friction and mechanical stress at the blade/obstacle interface, thereby improving the drilling performance. Although further studies with various parameters, including blade angle, number of blades, and pitch distance between blades are required to optimize the design of the MDD, this study shows a facile and rapid manufacturing method to fabricate biomedical microdevices using the additive manufacturing process.

## Conclusions

In summary, this study demonstrated the feasibility of a facile and rapid manufacturing method to fabricate micro-drilling devices using FFF-type 3D printing technology. Using the characteristics of the 3D-printed mold, precise and uniform blades were successfully fabricated for the drilling device. Drilling devices with three different blade shapes (45°, 0°, and helical type) were tailored and compared with a drilling device without a blade (plain type). A simple casting method was utilized to fabricate these drilling devices without complicated machining tools such as a laser cutter, chemical etcher, or lathe. CFD simulations and drilling tests were conducted to estimate the drag force and pressure distribution around each device and characterize the blade shape effects. Results showed that the microscale drilling device with 45° blades exhibited the best drilling performance with low fluidic resistance and the longest drilling depth, compared to other drilling devices. At a rotational speed of 1000 rpm, the drilling device with 45° blades penetrated 3.64 mm depth of the artificial 0.6% thrombus, whereas the device with conventional helical blades drilled 1.51 mm. This study demonstrates the use of additive manufacturing to fabricate microscale drilling devices for various biomedical and environmental applications.
